# Optical fibre taper-enabled waveguide photoactuators

**DOI:** 10.1038/s41467-022-28021-4

**Published:** 2022-01-18

**Authors:** Jianliang Xiao, Tao Zhou, Ni Yao, Shuqi Ma, Chenxinyu Pan, Pan Wang, Haoran Fu, Haitao Liu, Jing Pan, Longteng Yu, Shipeng Wang, Wenzhen Yang, Limin Tong, Lei Zhang

**Affiliations:** 1 Research Center for Intelligent Sensing, Zhejiang Lab, Hangzhou, 311100 China; 2 Institute of Flexible Electronics Technology of THU, Zhejiang Jiaxing, 314000 China; 3 State Key Lab of Modern Optical Instrumentation, College of Optical Science and Engineering, Zhejiang University, Hangzhou, 310027 China

**Keywords:** Actuators, Optical materials and structures

## Abstract

Photoactuators have attracted significant interest for soft robot and gripper applications, yet most of them rely on free-space illumination, which requires a line-of-site low-loss optical path. While waveguide photoactuators can overcome this limitation, their actuating performances are fundamentally restricted by the nature of standard optical fibres. Herein, we demonstrated miniature photoactuators by embedding optical fibre taper in a polydimethylsiloxane/Au nanorod-graphene oxide photothermal film. The special geometric features of the taper endow the designed photoactuator with microscale active layer thickness, high energy density and optical coupling efficiency. Hence, our photoactuator show large bending angles (>270°), fast response (1.8 s for 180° bending), and low energy consumption (<0.55 mW/°), significantly exceeding the performance of state-of-the-art waveguide photoactuators. As a proof-of-concept study, one-arm and two-arm photoactuator-based soft grippers are demonstrated for capturing/moving small objects, which is challenging for free-space light-driven photoactuators.

## Abstract (web summary)

Despite promising devices, waveguide photoactuators actuating performances have been fundamentally restricted by the nature of standard optical fibres. To overcome these challenges, authors propose an optical fibre taper-enabled waveguide photoactuator and show enhanced performance.

## Introduction

Soft actuators, which are capable of converting external stimuli to the desired shape changes or mechanical movements, can provide safe, comfortable and flexible human-machine interaction^1^, and hold great potential in many cutting-edge applications, such as soft robots^2–5^, artificial muscles^6–8^, biomimetic propellers^9–12^ and soft grippers^13–15^. Various soft actuators driven by different stimuli including magnetic fields^16,17^, electric fields^1,18,19^, temperature^20,21^, humidity^15,22^ and light^23–25^ have been widely reported. Among them, light-driven actuators, i.e., photoactuators have attracted particular research interest due to the advantages of light, such as immunity of electromagnetic interference, safe operation in explosive atmosphere and flexible tunability in wavelengths, intensity and polarization^23–27^.

To date, most photoactuators are triggered by free-space illumination, which requires a line-of-site low-loss optical path between the light source and the actuator^28–30^. As such, the utility of these photoactuators is vitally restricted in cases where direct and constant line-of-site access is absent. Besides, the intensity of free-space light may decrease significantly during long-distance transport, especially for the environment with strong absorption and scattering^9,13^. Moreover, for the free-space light-driven actuators to execute locomotion or handle objects, the control light generally needs to intentionally follow the motion of the actuator^1^, which vastly restricts the manoeuvrability and convenience. Alternatively, using optical fibre as waveguide to guide light into actuators provides an efficient strategy to overcome these limitations, since light can be transmitted over a long distance with low optical loss through flexible and bendable optical fibres^31^. In this strategy, optical fibres are combined with common photo-responsive materials to form a new kind of photoactuator, *i.e*. waveguide photoactuators^31–33^. For example, Zhou et al. reported a poly(N-isopropylacrylamide) waveguide photothermal actuator, which employed a plastic optical fibre to guide 532-nm-wavelength laser. For this photoactuator, the bending angle was <60° and the response time was over 50 s^32^. Kuenstler et al. demonstrated a waveguide photoactuator made of liquid crystal elastomer, achieving a bending angle of 15° and response time of longer than 5 s^31^. These reports shed light on the development of waveguide photoactuators, however, all of them suffered from limited bending amplitude and long response time. This could be ascribed to three possible reasons: (1) large thickness of the active layer due to the relatively large size of the commercially available optical fibre (typically >100 μm in diameter); (2) low energy density due to the non-focused light beam; (3) low optical coupling efficiency due to the size mismatch between the optical waveguide and the photo-responsive material. To address these issues, a thinner optical fibre with enhanced optical intensity is highly desired for high-performance photoactuators with large deformation and fast response.

Optical fibre tapers (OFTs), which are taper-drawn from standard optical fibres^34,35^ and have a tip diameter <1 μm, are capable of guiding light with tailorable optical confinement and low optical loss (e.g., <0.05 dB/cm^36^, much lower than any other optical waveguides of similar sizes). Therefore, the small size of OFT makes it possible to reduce the thickness of active layer and overcome the size mismatch between optical waveguide and photo-responsive material. More importantly, the energy density of light guided by OFT is much higher than that of commercially available optical fibres, resulting in a higher photothermal conversion efficiency.

**Fig. 1:**
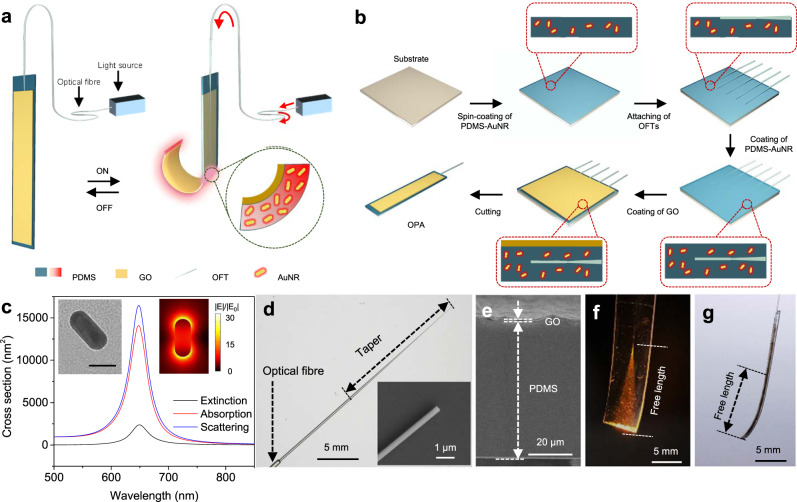
Structure, mechanism and fabrication of OPAs. **a** Schematic of the structure and driving mechanism of the OPA. **b** Preparation process of OPAs. **c** Calculated extinction, absorption and scattering cross section of an AuNR. The insets present a transmission electron microscope image of an AuNR (left) and a calculated near-field distribution of an AuNR at the wavelength of 638 nm (right). Scale bar: 30 nm. Source data are provided as a Source Data file. **d** Optical microscope image of an OFT with a taper length of ~1.75 cm. The inset presents a scanning electron microscopy image of the tip of the OFT showing a diameter of ~700 nm. **e** Cross-sectional view of the PDMS-GO film. The thicknesses of PDMS and GO layer are ~70 and 1.5 μm, respectively. **f** Photograph of sending 635 nm light into an OPA with a broadened width to show the light propagation path. **g** Photograph of an OPA with a typical size of ~500 μm in width and ~1 cm in free length.

Herein, we propose an OFT photoactuator (OPA) that can drive large deformation, in which OFT is embedded in a photo-responsive photothermal film and used as a key component to guide light into the photothermal film (Fig. 1a). Benefiting from the special geometric features of OFT, i.e., micro/nanoscale diameter and long taper region, the OPA features a thin active layer, high energy density and optical coupling efficiency, which enable a much larger bending angle (>270°) than that of waveguide actuators reported before, high actuation efficiency and low energy consumption (<0.55 mW/°). In addition, the OPA demonstrates excellent stability, controllability and manoeuvrability during repeated actuation, which is particularly valuable for accurate control when executing a task that requires dynamic location. As an example, we show that OPA soft gripper can capture, move and release objects with different shapes at a wide operating distance, which is challenging for free-space light-driven actuators. We believe that OPA will offer an effective strategy to design easy-to-manoeuvre photoactuators with large deformation.

## Results

### Fabrication of OPAs

As shown in Fig. 1a, an OPA is composed of a photo-responsive elastomeric composite sheet with an OFT embedded in the middle as the waveguide. The elastomeric composite sheet is made of a polydimethylsiloxane (PDMS) film, which is doped with Au nanorods (AuNRs) as the photothermal agent, and a graphene oxide (GO) film. When a control light is launched into the elastomeric composite via the OFT, photothermal heating induced by the AuNR will cause a significant expansion of the PDMS/AuNR layer due to the high coefficient of thermal expansion (CTE) of PDMS^37,38^. On the other hand, GO layer undergoes negligible thermal expansion due to its low CTE. Thus, the mismatch between the CTE and deformations of two layers leads to a dramatic bending of the OPA. When the light is switched off, the OPA recovers back to its initiate state. Figure 1b shows a detailed preparation process of the OPA. Firstly, a thin layer of PDMS doped with AuNRs was spin-coated onto a glass substrate as the first layer of the elastomeric composite sheet. Then, an array of OFTs were embedded in the PDMS layer with an interval of ~5 mm, which were subsequently covered with a thin layer of GO sheet. Finally, as fabricated photo-responsive elastomeric composite sheet was cut into small pieces to obtain OPAs. AuNRs used in OPAs, with average length and diameter of ~51 and 23 nm respectively (Fig. 1c, left inset), show an ensemble longitudinal surface plasmon resonance peak around 638 nm (Supplementary Fig. 1), which matches well with the peak wavelength of the control light. Due to the high absorption cross section (Fig. 1c) and strong local-field enhancement (Fig. 1c, right inset), the AuNRs can efficiently generate heat in the PDMS layer via the nonradiative decay of LSPR excited by the control light. OFTs were fabricated by flame-heated taper drawing^34,35^ of standard optical fibres. Figure 1d shows an optical microscope image of a typical OFT with a taper length of ~1.75 cm and a tip diameter of ~700 nm. An optical microscope image of OFT embedded in a PDMS-AuNR film is shown in Supplementary Fig. 2. Similar to the OFT shown in Fig. 1d, the embedded OFT has a diminishing diameter from the undrawn optical fibre part (125 μm) to the taper and waist region (~700 nm). The relatively long taper can effectively suppress the intermodal energy transfer in the taper region and satisfy the adiabatic condition^34,39^, guaranteeing the high energy efficiency, while the small tip diameter is advantageous for the seamless shrink of beam size down to micrometre scale. The cross-sectional view of an as-fabricated elastomeric is shown in Fig. 1e, in which a layer of PDMS/AuNR with a thickness of ~70 μm and a layer of GO with a thickness of ~1.5 μm can be clearly seen. Despite of the thin thickness, the GO layer shows a fine lamellar structure and typical wrinkled surface, benefiting from the outstanding self-assembly ability of GO sheets with large lateral dimensions (Supplementary Fig. 3). When guiding a 635 nm-wavelength laser into an OPA, a clear light propagation path in the sheet can be observed due to the scattering of light by the AuNRs (Fig. 1f). The width of the OPA in Fig. 1f was designedly broadened to completely display the light propagation path, while the typical size of an OPA is usually ~500 μm in width (Supplementary Fig. 4) and ~1 cm in free length (Fig. 1g).

### Light-driven actuation

**Fig. 2:**
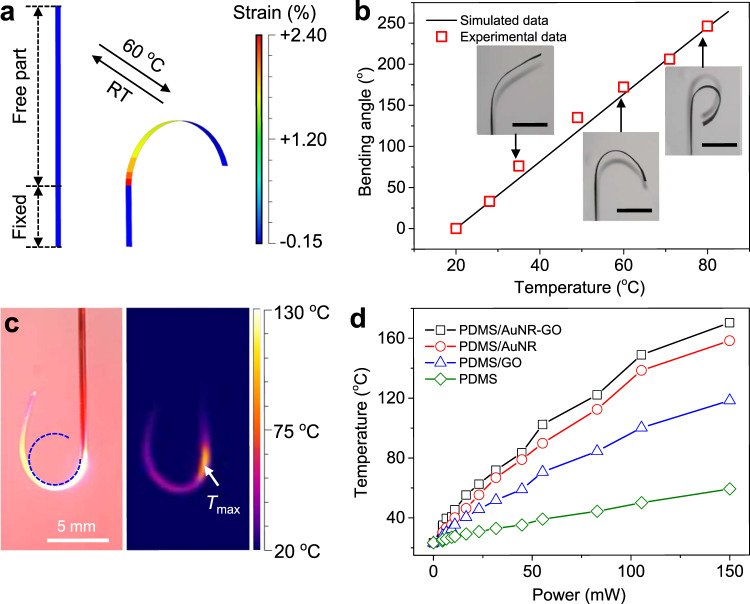
Deformation and photothermal heating of OPA. **a** Finite element analysis of the OPA model (10 × 0.5 × 0.07 mm^3^) by applying homogeneous temperature on the structure. **b** Simulated and experimental thermal bending angles of the OPA (10 × 0.5 × 0.07 mm^3^) under different temperature. Scale bar: 5 mm. Source data are provided as a Source Data file. **c** Photograph (left) and infrared thermography image (right) of the OPA under 635 nm laser of 75 mW. The blue dotted line helps to show a diminishing bending curvature along the longitudinal direction of the OPA. **d** Measured *T*_max_ of the OPA with various components as a function of the laser power. Source data are provided as a Source Data file.

As previously mentioned, the actuation mechanism of OPA is based on the CTE mismatch and asymmetric deformation of the PDMS/AuNR-GO bilayer under photothermal heating. We first investigated the deformation behaviour of an OPA when a temperature rise is directly applied onto the bilayer structure. As shown in Fig. 2a, the PDMS/GO bilayer (70 μm/1.5 μm) undergoes a reversible bending when the environmental temperature is switched between room temperature (RT, 20 °C) and 60 °C, which is simulated based on finite element analysis (FEA, see details in Methods). The simulated bending angle of the OPA increases linearly with the increase of environmental temperature (Fig. 2b and Supplementary Fig. 5), which agrees very well with the experimental results by putting it on a hot plate. Thus, by taking advantage of the photothermal effects of embedded AuNRs, OPA can undergo similar bending. As shown in Fig. 2c, when a 635 nm-wavelength laser (75 mW) was launched into the OPA, the temperature rise induced by the photothermal effect leads to a significant bending of OPA, with the highest temperature (*T*_max_) of ~110 °C located near the OFT tip of the OPA. It is worth to note that there is a diminishing bending curvature along the longitudinal direction due to the gradient distribution of temperature. Figure 2d compares laser power-dependent *T*_max_ of OPAs fabricated with different components. As expected, the AuNR plays an important role in the light-to-heat conversion of the OPA, and the GO also shows notable conversion ability due to its light adsorption property in the UV–Vis spectral range (Supplementary Fig. 6). The *T*_max_ of an OPA composed of PDMS/AuNR-GO under 150 mW laser is as high as 170 °C, which is benefited from the significantly enhanced energy density near the OFT tip and the high photothermal conversion efficiency of AuNRs.

**Fig. 3:**
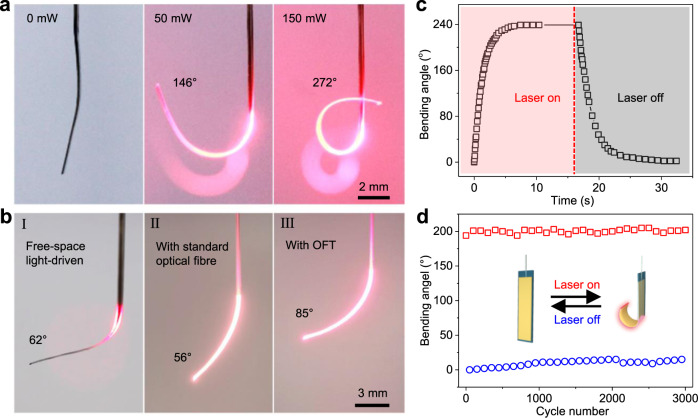
Light-driven actuating performances of OPA. **a** Photographs showing the light-driven bending of an OPA (10 × 0.5 × 0.07 mm^3^) under different laser powers. **b** Free-space light-driven bending of the OPA (I), waveguided light-driven bending of an actuator (thickness of ~220 μm) fabricated using a standard optical fibre (II), and waveguided light-driven bending of OPA with a thickness of ~225 μm (III) under 635 nm-wavelength laser (150 mW). **c** Light-driven bending of an OPA (the same as in **a**) exposed to 635 nm-wavelength laser as a function of time. Source data are provided as a Source Data file. **d** Bending angles of the OPA over 3000 cycles of on-off switch of laser with a power of 100 mW. Source data are provided as a Source Data file.

The light-driven deformation amplitude of OPAs can be readily regulated by controlling the laser power. With the increase of control laser power, the bending angle increases gradually (Fig. 3a and Supplementary Fig. 7). The highest bending angle is larger than 270° at 150 mW, which is much larger than the bending angle (<60°) of other types of waveguide actuators^31–33^. In addition, the bending angle of OPA is also much larger than that of the actuator driven by free-space light under the same laser power of 150 mW (Fig. 3b, I). When illuminated with a free-space laser beam, the PDMS/AuNR-GO film can only absorb a small portion of the light, while the rest is wasted since the illuminating area is larger than the film. As a result, the energy efficiency is pretty low and the bending angle is only 62°. Using OFT to guide light into the PDMS/AuNR-GO film can effectively overcome this problem since the light can be guided along the film and absorbed continuously, allowing for higher energy efficiency and larger bending angle. The energy consumption to drive the OPA is as low as <0.55 mW/°, indicating the high actuation efficiency with the use of OFT, which is favourable for the cost-cutting and miniaturization of the integrated device in practical applications.

On the other hand, the small tip diameter of the OFT (Fig. 1d) allows for reducing the thickness of OPA to <100 μm, which is favourable for large bending angles. As a contrast, the total thickness of an actuator fabricated with a standard optical fibre (125 μm in diameter) has to be ~220 μm to guarantee the stable embedding of the optical fibre in the PDMS-AuNR matrix (Supplementary Fig. 8a, b), and as a result the bending angle under 150 mW laser is as small as 56° (Fig. 3b, II). The detailed influences of thicknesses of each layer on the bending angles of OPA are shown in Supplementary Fig. 9. In addition, an OPA with a similar thickness of ~225 μm (Supplementary Fig. 8c), which was intentionally designed, also shows larger bending angle (85°) than that of standard optical fibre-based actuator (Fig. 3b, III). This indicates that the enhanced energy density, produced by the small tip diameter of the OFT, also favours the efficient actuation of the OPA. Besides, optical fibre with a diameter of 80 μm was used to fabricate actuators with different thicknesses (Supplementary Fig. 10). As shown in Supplementary Fig. 11a, when the total thickness of actuator is ~100 μm, the optical fibre pierces the thin PDMS layer, causing a leakage of light and reduced bending angle (80°). Increasing the thickness to ~120 μm can effectively improve the stability of actuator, while the bending angle (118°, Supplementary Fig. 11b) is much smaller than that of OPA with a similar thickness (~150°, Supplementary Fig. 9), which is also ascribed to the enhanced energy density of OFT. In summary, the thickness as thin as 70 μm, along with the improved energy density and optical coupling efficiency, endow the OPA with excellent light-driven actuating performances.

Dynamic deformation of the OPA under 120 mW laser indicates a rapid response of the OPA (Fig. 3c and Supplementary Movie 1). It takes only 1.8 s to reach a bending angle >180° (~70% of the total deformation), whereas the recovery of the 70% bending takes 2.3 s after the switch off of the laser. This is understandable because the cooling process of PDMS is slower than the heating process. The fast response of OPAs is probably due to the high energy density and thin thickness of the active layer benefiting from the small tip of OPT. The bending force of an OPA under 150 mW laser was measured to be ~400 μN (Supplementary Fig. 12). The actuating performances of OPAs, including bending angle, energy consumption and response time, surpass that of all previously reported waveguide photoactuators and many free-space light-driven photoactuators (Supplementary Table 1).

To evaluate the working stability of OPAs, their tensile properties were firstly tested. As shown in Supplementary Fig. 13 and Supplementary Movie 2, the GO layer of an OPA breaks at a strain of ~5.2 %, causing a sharp decrease of tensile stress in the tensile stress-strain curve. In addition, optical microscope images of an OFT embedded in PDMS-AuNR under different strain (Supplementary Fig. 14) show that the seamless connection between the OFT and the PDMS-AuNR matrix is not destroyed under a strain of 20 %, indicating a high stability of the interface between OFT and PDMS-AuNR matrix. In another word, during stretching deformation, the OPA will be damaged under tensile strain larger than 5% due to the breaking of GO layer rather than the delamination between the OFT and the PDMS-AuNR matrix. Further increasing the tensile force to ~8 N causes the breaking of the OFT in the PDMS-AuNR matrix, followed by the pulling out of the OFT with a corresponding delaminating force of ~6 N (Supplementary Fig. 15).

On the other hand, given that the main deformation of an OPA during working is from the bending of the free part, the working stability of an OPA largely relies on the variation of bending angle during the cyclic bending/recovery process. As shown in Fig. 3d and Supplementary Fig. 16, the OPA shows negligible decay in deformation after more than 3000 actuations with a standard deviation of 3.62° and coefficient of variation as low as 0.14, verifying the outstanding working stability and durability of the OPA. The excellent stability and manoeuvrability endow OPAs with significant advantages in accurate control when executing a task that involves location changes, while the free-space light-driven actuators suffer from the difficulties in accurately controlling the illuminating spot.

### OPA grippers for handling objects

Target capturing/moving is an important and challenging application of photoactuators. For the use of free-space light-driven actuators to capture/move objects, the illuminating spot needs to be accurately controlled to follow the moving of actuators or a large illuminating beam covering the whole operating area should be supplied, which greatly restricts the manoeuvrability and operating area. However, for OPAs demonstrated here, they are seamlessly connected to optical fibres, allowing the control light to intrinsically follow the moving of actuators. Thus, OPAs hold significant advantages in accurately handling and moving objects in a wide operating area.

**Fig. 4:**
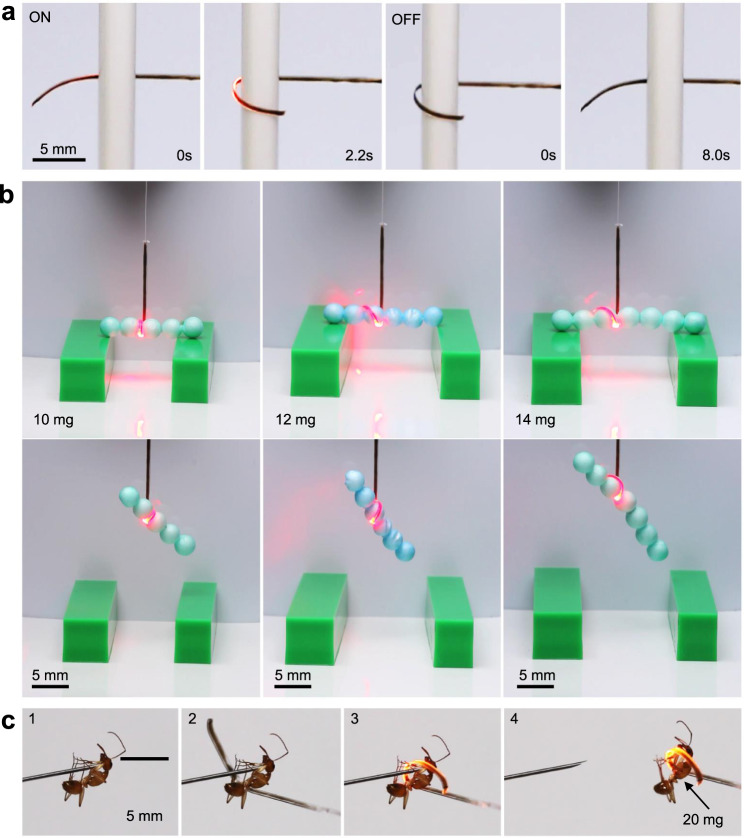
One-arm OPA gripper captures objects. **a** Photographs of winding and unwinding a pipe with an OPA. **b** Photographs of capturing glued balls of different weights with a one-arm gripper. **c** Photographs of capturing an ant with a one-arm gripper.

The large bending angle enables an OPA to wrap around and capture small objects. As shown in Fig. 4a and Supplementary Movie 3, the OPA winds rapidly onto a plastic pipe under 635-nm laser within 2.2 s (panels 1 and 2), and unwinds gradually after switching the control laser off (panels 3 and 4). Similarly, as shown in Fig. 4b and Supplementary Movies 4–6, the OPA acting as a one-arm gripper can capture and lift glued balls, with a maximum lifting weight of ~14 mg. Although the capture of 16 mg balls was failed (Supplementary Fig. 17a and Supplementary Movie 7), a heavier target with more complex shape can be captured and lifted by adjusting the angle for capturing and taking advantage of the OFT to supply an additional supporting force. As shown in Fig. 4c and Supplementary Movie 8, the one-arm OPA gripper can grasp an ant (~20 mg) stayed on a tip immediately with the switch on of control laser and then take it off from the tip.

**Fig. 5:**
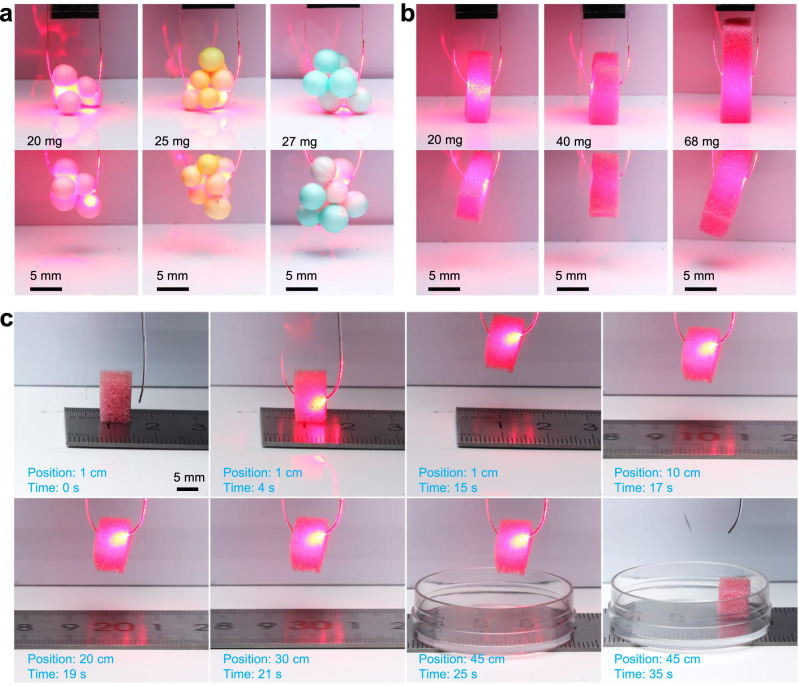
Two-arm OPA soft gripper captures and moves objects. **a** Photographs of capturing glued balls of different weights with a two-arm OPA gripper. **b** Photographs of capturing cuboids of different weights with a two-arm OPA gripper. **c** Photographs of handling a cuboid in a long operating distance with a two-arm OPA gripper fixed on an electric slider.

Furthermore, a two-arm OPA soft gripper was fabricated to capture objects with different shapes. As shown in Fig. 5a and Supplementary Movies 9–11, the two-arm OPA gripper can capture and lift a group of small balls with a maximum weight of 27 mg. Using sponge cuboids with rougher surface as targets, the maximum lifting weight of the two-arm OPA gripper can be further increased to 68 mg (Fig. 5b and Supplementary Movies 12–14). This is because the rougher surface increases the friction coefficient, which favours the capturing capacity^40^. The weight of free parts of the two-arm OPA gripper is ~0.95 mg, thus the corresponding lifting weight ratio is ~71, which is higher than that of many previously reported photoactuaors (Supplementary Table 1). With the further increase of the weight of object, the OPA gripper failed to capture, while the structure and functions of OPAs were not destroyed (Supplementary Fig. 17b, c and Supplementary Movies 15 and 16).

Two-arm OPA soft gripper can also pick up, transfer (with a speed of ~50 mm/s) and release a cuboid into a container ~44 cm far away when it was fixed on an electric slider, as shown in Fig. 5c and Supplementary Movie 17. These results demonstrate the advantages of OPA in accurately handling objects in a wide operating area, while free-space light-driven actuators suffering from the difficulties in accurately controlling the illuminating spot to follow the moving of actuators during operation.

## Discussion

In summary, we have demonstrated miniature waveguide photoactuators (i.e. OPAs) using OFT to guide the control light, which are free from the restrictions of free-space light. The special geometric features of the OFT endow the OPA with much enhanced actuating performances, overcoming the main challenges to develop waveguide photoactuators with large bending amplitude, and thus offering an effective strategy design photoactuators driven by waveguided light. Differently from the free-space light-driven actuators which suffer from the difficulties in accurately controlling the illuminating spot, the light supply of OPA is located inside the material and controlled in real time, which gives OPAs significant advantages in executing tasks involving location changes, such as handling and moving objects with different shapes. The more complex deformation of OPAs can be readily achieved by regulating the transport and absorption of light in it and more potential applications, such as searching in unstructured environment, deepwater sampling, and in vivo diagnosis/therapy, can be realized by the special designs of the deformation behaviours and the implementation of advanced materials.

## Methods

### Fabrication of OPA strips and grippers

AuNRs were prepared using a seed-mediated method^41^. Briefly, the seed solution was prepared by the addition of an ice-cold sodium borohydride solution (0.6 mL, 0.01 M) into a mixture solution composed of chloroauric acid (0.25 mL, 0.01 M) and cetyltrimethylammonium bromide (CTAB, 9.75 mL, 0.1 M). The resultant solution was vigorously stirred for 2 min and then kept at 25 °C for 2 h before use. The growth solution was prepared by the sequential addition of chloroauric acid (2 mL, 0.01 M), silver nitrate (0.4 mL, 0.01 M), ascorbic acid (0.32 mL, 0.1 M), and hydrochloric acid (0.8 mL, 1.0 M) into a CTAB aqueous solution (40 mL, 0.1 M). 8 μL seed solution was then added into the growth solution. The resultant solution was gently stirred for 20 s and left undisturbed overnight, and then centrifuged at 14,000 g for 10 min, decanted, and resuspended in water to remove excess CTAB. Gold nanorods were then modified with a monolayer of polyethylene glycol (PEG) by adding mPEG5000-SH (200 μL, 5 mM) into gold nanorod solution (1 mL, 1 mM). The mixture was stirred for 24 h at room temperature, and then cleaned by centrifuge. Then the as-prepared PEGylated AuNRs dispersed in ethanol were mixed with PDMS prepolymer component A (5 g, SYLGARD 184, DOW) before adding the component B (0.5 g). Then the degassed PDMS/AuNR prepolymer was spin-coated on a glass substrate pretreated with plasma, followed by curing at 80 °C for 30 min. OFTs were fabricated from standard optical fibres (MMF 62.5/125, Corning) based on the flame brushing method^34,35,42,43^. Briefly, a standard optical fibre was heated by an acetylene flame with a flame width of 5 mm and pulled up to break with a relative high velocity of 150-300 mm/s. The taper length and tip diameter of OFT could be modulated by controlling the pulling velocity. The obtained OFTs were then fixed on the as-prepared PDMS film, on which the PDMS/AuNR prepolymer was casting, and two strips of polyimide tape were used as spacers to control the thickness of PDMS films. After curing at 80 °C for 30 min, PDMS/AuNR films with OFTs embedded in were peeled off from the substrate and treated with plasma to improve the hydrophilicity, followed by coating of GO suspensions (3 mg/mL, GaoxiTech.). The sample was then dried at 50 °C and cut into individual strips containing single OFT, getting OPA strips. Due to the internal stress introduced during curing, the PDMS film showed a pre-bending after peeling off from the substrate, while the bending angle depended on the thickness and heating conditions. The pre-bending direction of obtained OPA strip could be adjusted by choosing the side to coat GO. The thickness of PDMS and GO layer in an OPA strip was regulated by the speed of spin-coating, thickness of polyimide tape spacer, and the volume of GO suspension. For the fabrication of OPA grippers, two OPA grippers were face-to-face attached on a cuboid with a gap of 1 cm and free-standing length of 1.5 cm.

### Characterization and measurements

The morphology of an OPA was characterized by optical microscope (Nikon, LV150N) and field-emission scanning electron microscopy (SEM, S4800, Hitachi). UV–Vis absorption spectra were measured on a UV–Vis spectrophotometer (CARY100, Varian). The temperature of an OPA under different laser powers was measured by an infrared camera (T8, Dali Tech.). Simulated light transport in OFT was performed using Rsoft BeamPROP. The bending force was measured using a nano-force sensor (NFS-B, Nators). Tensile tests were carried out on a universal testing machine (ESM303, Mark-10) at room temperature. The specimens were fixed according to the methods demonstrated in Supplementary Fig. 19. The testing displacement rate was 0.5 mm/min.

### Actuation analysis of the OPA

The actuation behaviours of the OPA strips and grippers were captured by a digital video camera. The bending angles were calculated based on the method demonstrated in Supplementary Fig. 20. Thermal actuation was measured using a temperature-programmed heating plate. Light-driven actuation was achieved under 20 °C using a continuous-wave laser (OX-6351, Oxlasers) with wavelength of 635 nm and maximum power of 1 W.

### FEA simulations

The commercial finite-element analysis software ABAQUS was used for the stress analysis of thermal actuation, employing the Abaqus/General solver. The thicknesses of PDMS (Young’s modulus 1 MPa, Poisson’s ratio 0.48, thermal expansion 300 × 10^−6^ K^−1^) layer and GO (Young’s modulus 30 GPa, Poisson’s ratio 0.1, thermal expansion 0.85 × 10^−6^ K^−1^) layer were 70 and 1.5 μm, respectively. The predefined field of temperature was applied to fulfill the computation of thermal deformation from 20 °C to 85 °C.

## Supplementary information

Supplementary Information

Description of Additional Supplementary Files

Supplementary Movie 1

Supplementary Movie 2

Supplementary Movie 3

Supplementary Movie 4

Supplementary Movie 5

Supplementary Movie 6

Supplementary Movie 7

Supplementary Movie 8

Supplementary Movie 9

Supplementary Movie 10

Supplementary Movie 11

Supplementary Movie 12

Supplementary Movie 13

Supplementary Movie 14

Supplementary Movie 15

Supplementary Movie 16

Supplementary Movie 17

## Data Availability

Source data underlying Figs. 1c, 2b, c, 3c, d, and Supplementary Figs. 1, 6, 7, 9, 12, 13 and 15 are provided as a Source Data file with this paper. All other relevant data are available from the corresponding author on reasonable request. Source data are provided with this paper.

## Source data

Source Data

## References

[1] Xiao YY, Jiang ZC, Tong X, Zhao Y (2019). Biomimetic locomotion of electrically powered “Janus” soft robots using a liquid crystal polymer. Adv. Mater..

[2] Hu W, Lum GZ, Mastrangeli M, Sitti M (2018). Small-scale soft-bodied robot with multimodal locomotion. Nature.

[3] Gu G, Zou J, Zhao R, Zhao X, Zhu X (2018). Soft wall-climbing robots. Sci. Robot..

[4] Rich SI, Wood RJ, Majidi C (2018). Untethered soft robotics. Nat. Electron..

[5] Palagi S, Fischer P (2018). Bioinspired microrobots. Nat. Rev. Mater..

[6] Han B (2019). Plasmonic-assisted graphene oxide artificial muscles. Adv. Mater..

[7] Matsuda T, Kawakami R, Namba R, Nakajima T, Gong JP (2019). Mechanoresponsive self-growing hydrogels inspired by muscle training. Science.

[8] Zhu QL (2020). Light-steered locomotion of muscle-like hydrogel by self-coordinated shape change and friction modulation. Nat. Commun..

[9] Shahsavan H (2020). Bioinspired underwater locomotion of light-driven liquid crystal gels. Proc. Natl Acad. Sci. USA.

[10] Li MT, Wang X, Dong B, Sitti M (2020). In-air fast response and high speed jumping and rolling of a light-driven hydrogel actuator. Nat. Commun..

[11] Fan W (2019). Dual-gradient enabled ultrafast biomimetic snapping of hydrogel materials. Sci. Adv..

[12] Zeng H (2019). Light-fuelled freestyle self-oscillators. Nat. Commun..

[13] Pilz da Cunha M, Kandail HS, den Toonder JMJ, Schenning APHJ (2020). An artificial aquatic polyp that wirelessly attracts, grasps, and releases objects. Proc. Natl Acad. Sci. USA.

[14] Wani OM, Zeng H, Priimagi A (2017). A light-driven artificial flytrap. Nat. Commun..

[15] Dong Y (2019). Multi-stimuli-responsive programmable biomimetic actuator. Nat. Commun..

[16] Alapan Y, Karacakol AC, Guzelhan SN, Isik I, Sitti M (2020). Reprogrammable shape morphing of magnetic soft machines. Sci. Adv..

[17] Deng H (2020). Laser reprogramming magnetic anisotropy in soft composites for reconfigurable 3D shaping. Nat. Commun..

[18] Leroy E, Hinchet R, Shea H (2020). Multimode hydraulically amplified electrostatic actuators for wearable haptics. Adv. Mater..

[19] Ji X (2019). An autonomous untethered fast soft robotic insect driven by low-voltage dielectric elastomer actuators. Sci. Robot..

[20] Downs FG (2020). Multi-responsive hydrogel structures from patterned droplet networks. Nat. Chem..

[21] Jin B (2018). Programming a crystalline shape memory polymer network with thermo- and photo-reversible bonds toward a single-component soft robot. Sci. Adv..

[22] Zhang YL (2020). A “Yin”-“Yang” complementarity strategy for design and fabrication of dual-responsive bimorph actuators. Nano Energy.

[23] Li J, Zhou X, Liu Z (2020). Recent advances in photoactuators and their applications in intelligent bionic movements. Adv. Opt. Mater..

[24] Li Y, Liu Y, Luo D (2020). Polarization dependent light-driven liquid crystal elastomer actuators based on photothermal effect. Adv. Opt. Mater..

[25] Gelebart AH (2017). Making waves in a photoactive polymer film. Nature.

[26] Ge F, Yang R, Tong X, Camerel F, Zhao Y (2018). A Multifunctional dye-doped liquid crystal polymer actuator: light-guided transportation, turning in locomotion, and autonomous motion. Angew. Chem., Int. Ed..

[27] Pang X, Lv J, Zhu C, Qin L, Yu Y (2019). Photodeformable azobenzene-containing liquid crystal polymers and soft actuators. Adv. Mater..

[28] Sitti M, Wiersma DS (2020). Pros and cons: magnetic versus optical microrobots. Adv. Mater..

[29] Hu Y, Li Z, Lan T, Chen W (2016). Photoactuators for direct optical-to-mechanical energy conversion: from nanocomponent assembly to macroscopic deformation. Adv. Mater..

[30] Zeng H, Wasylczyk P, Wiersma DS, Priimagi A (2018). Light robots: bridging the gap between microrobotics and photomechanics in soft materials. Adv. Mater..

[31] Kuenstler AS, Kim H, Hayward RC (2019). Liquid crystal elastomer waveguide actuators. Adv. Mater..

[32] Zhou Y, Hauser AW, Bende NP, Kuzyk MG, Hayward RC (2016). Waveguiding microactuators based on a photothermally responsive nanocomposite hydrogel. Adv. Funct. Mater..

[33] Zmyslony M (2020). Optical pliers: Micrometer-scale, light-driven tools grown on optical fibres. Adv. Mater..

[34] Yao N (2020). Ultra-long subwavelength micro/nanofibres with low loss. IEEE Photonics Technol. Lett.

[35] Xu Y, Fang W, Tong L (2017). Real-time control of micro/nanofibre waist diameter with ultrahigh accuracy and precision. Opt. Express.

[36] Nagai R, Aoki T (2014). Ultra-low-loss tapered optical fibres with minimal lengths. Opt. Express.

[37] Han B, Zhang YL, Chen QD, Sun HB (2018). Carbon-based photothermal actuators. Adv. Funct. Mater..

[38] Bowden N, Huck WTS, Paul KE, Whitesides GM (1999). The controlled formation of ordered, sinusoidal structures by plasma oxidation of an elastomeric polymer. Appl. Phys. Lett..

[39] Birks TA, Li YW (1992). The shape of fibre tapers. J. Lightwave Technol..

[40] Yang Y, Vella K, Holmes D (2021). Grasping with kirigami shells. Sci. Robot..

[41] Wang P (2015). Single-band 2-nm-line-width plasmon resonance in a strongly coupled au nanorod. Nano Lett..

[42] Zhang Z (2020). Optical micro/nanofibre embedded soft film enables multifunctional flow sensing in microfluidic chips. Lab Chip.

[43] Pan J, Zhang Z, Jiang C, Zhang L, Tong L (2020). A multifunctional skin-like wearable optical sensor based on an optical micro-/nanofibre. Nanoscale.

