# Supplementary material for: Optical fibre taper-enabled waveguide photoactuators
Source: Nat Commun. 2022 Jan 18;13:363. doi: 10.1038/s41467-022-28021-4 (PMC8766484; doi:10.1038/s41467-022-28021-4)
Supplement: Supplementary file 1 — Supplementary Information [file 41467_2022_28021_MOESM1_ESM.pdf]

Supplementary Information for

Optical fibre taper-enabled waveguide photoactuators

Jianliang Xiao[1], Tao Zhou[2], Ni Yao[1], Shuqi Ma[1], Chenxinyu Pan[3], Pan Wang*[3], Haoran Fu[2], Haitao Liu[1], Jing Pan[3], Longteng Yu[1], Shipeng Wang[1], Wenzhen Yang*[1], Limin Tong[3], Lei Zhang*[1,3]

[1] Research Center for Intelligent Sensing, Zhejiang Lab, Hangzhou 311100, China.

[2] Institute of Flexible Electronics Technology of THU, Zhejiang, Jiaxing 314000, China.

[3] State Key Lab of Modern Optical Instrumentation, College of Optical Science and Engineering, Zhejiang University, Hangzhou 310027, China.

Corresponding author:

*E-mail: nanopan@zju.edu.cn; ywz@zhejianglab.edu.cn; zhang_lei@zju.edu.cn

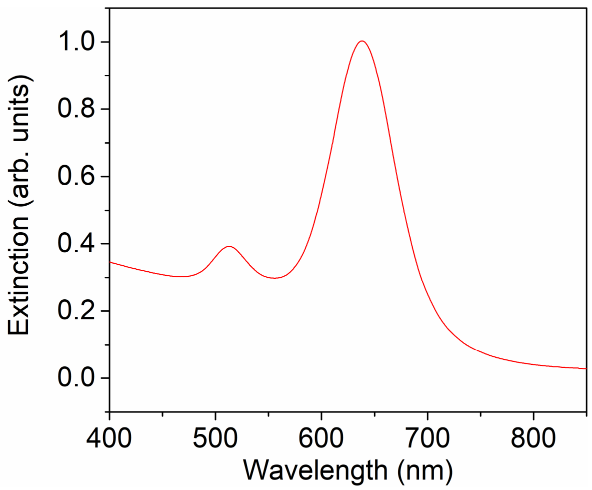

Supplementary Fig. 1 Extinction spectrum of AuNRs dispersed in water. Source data are provided as a Source Data file.

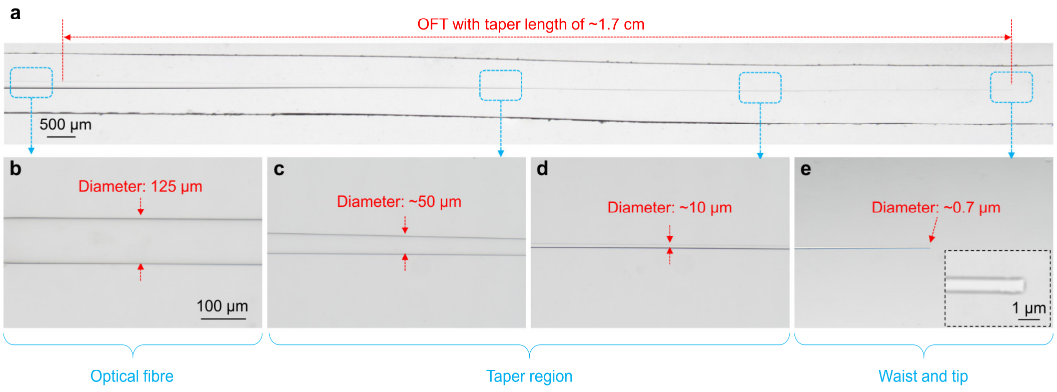

Supplementary Fig. 2 Optical microscope image of OFT embedded in PDMS-AuNR film. a Optical microscope image of an OPA without GO layer showing the total length of an OFT embedded in a PDMS-AuNR. **b-e** Optical microscope images of the OFT with high magnification taken at (**b**) the undrawn optical fibre region with a typical diameter of 125 μm, (**c**, **d**) the taper region with a diameter of ~50 μm and ~10 μm respectively, and (**e**) the waist and tip with a diameter of ~700 nm. Insert in **e** shows higher magnification image of the tip of OFT. The GO layer was removed on purpose to show the morphology of OFT more clearly.

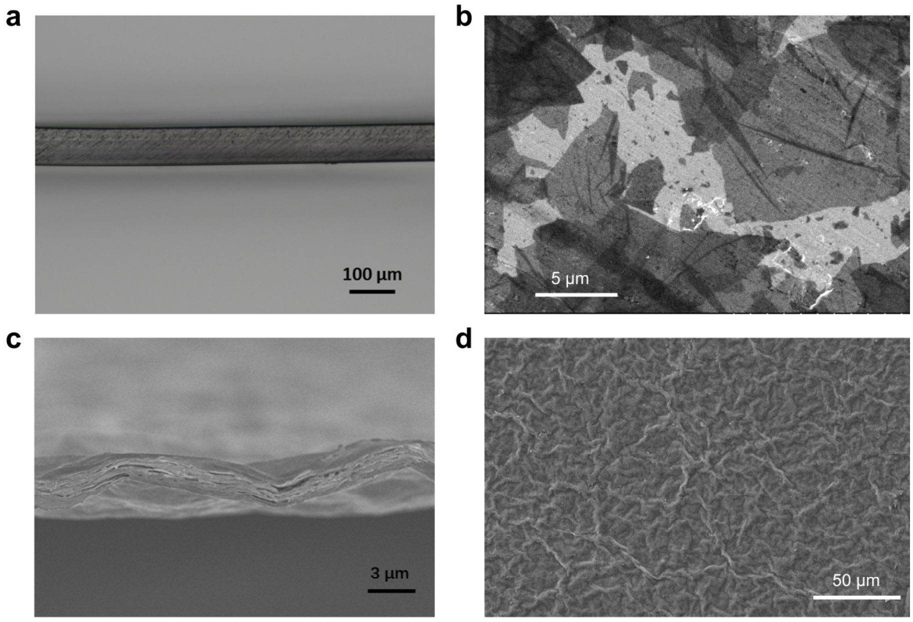

Supplementary Fig. 3 Morphology of OPA and GO layer. **a** Optical microscope image of the OPA showing a total thickness of ~70 μm. **b** Scanning electron microscopy image of GO sheets with a lateral size of 10-20 μm. **c** Scanning electron microscopy image of the section of GO film with a typical lamellar structure. **d** Scanning electron microscopy image of the surface of GO film.

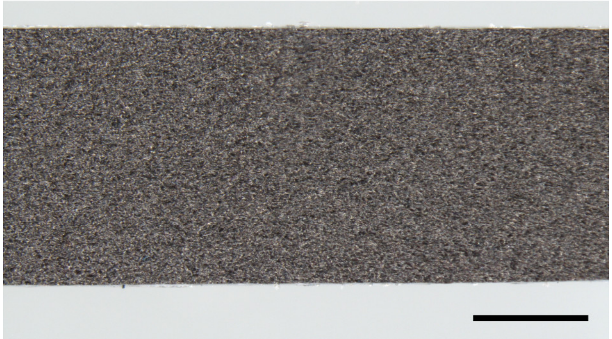

Supplementary Fig. 4 Optical microscope image of the free part of an OPA taken at the GO side. Scale bar: 100 μm. The typical width of OPA is ~500 μm.

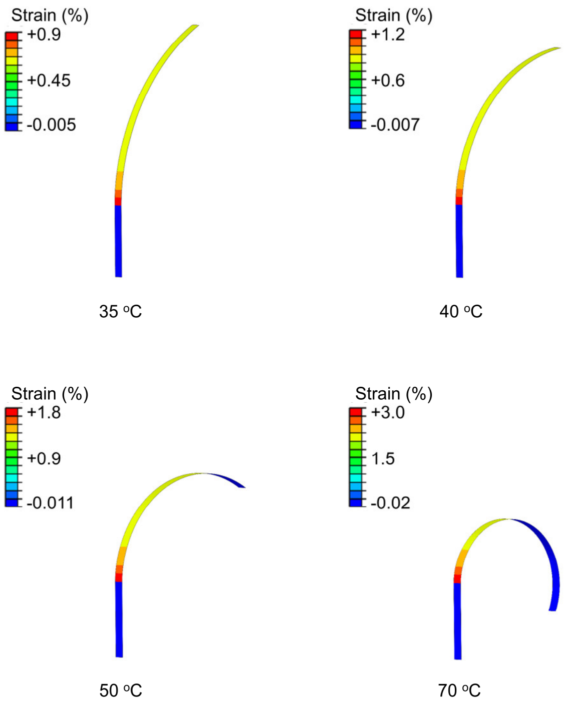

Supplementary Fig. 5 FEA of the OPA model ($10 \times 0.5 \times 0.07$ mm^3) by applying various homogeneous temperature on the structure.

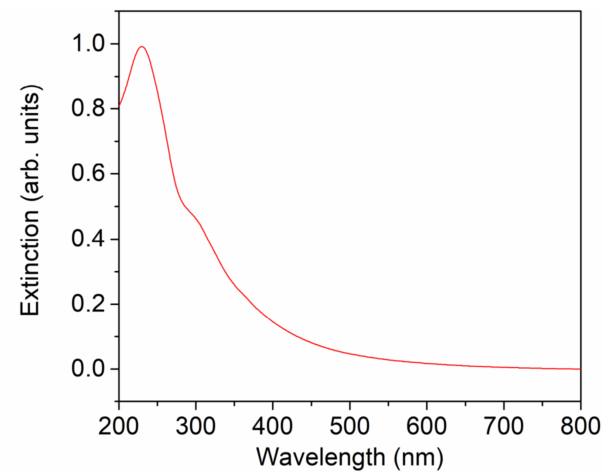

Supplementary Fig. 6 UV-Vis absorbance spectrum of GO. Source data are provided as a Source Data file.

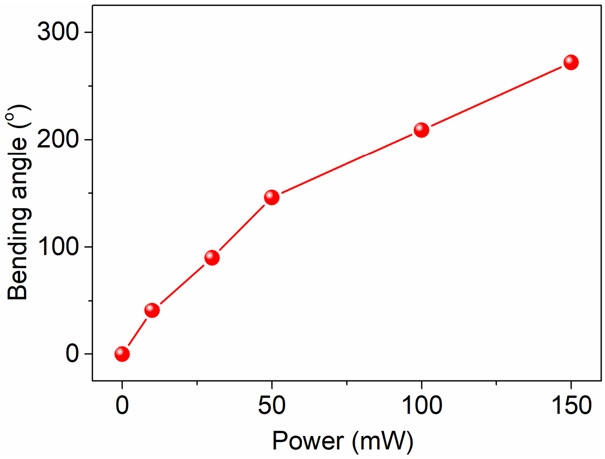

Supplementary Fig. 7 Light-driven bending angles of the OPA as a function of the laser power. Source data are provided as a Source Data file.

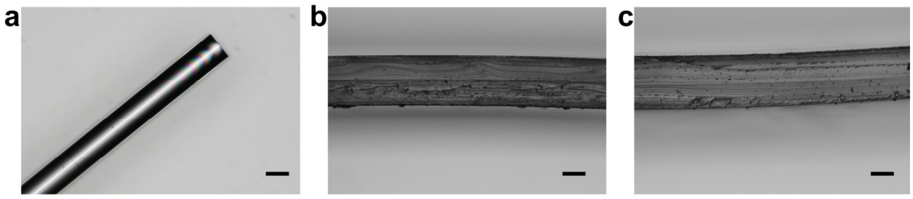

Supplementary Fig. 8 Optical microscope images of a standard optical fibre and actuators with large thickness.

a Optical microscope image of a standard optical fibre. **b** Optical microscope image of an actuator fabricated with a standard optical fibre showing a total thickness of ~220 µm. **c** Optical microscope image of an OPA with a total thickness of ~225 µm. Scale bar: 100 µm.

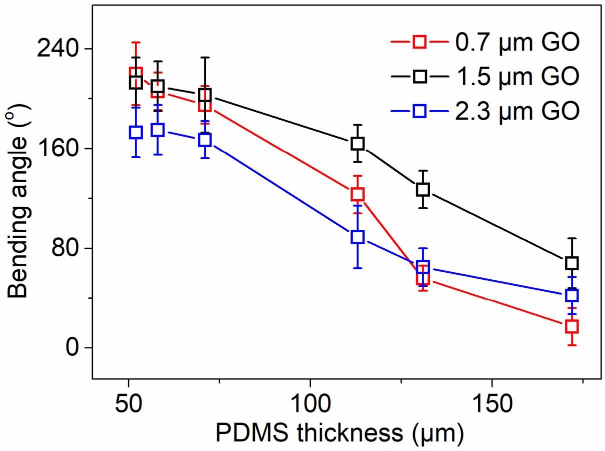

Supplementary Fig. 9 Light-driven bending angles of OPAs (10 × 0.5 mm^2) with various thicknesses of PDMS and GO layers under 635 nm laser of 100 mW. Error bars are s.d. from five samples. Source data are provided as a Source Data file.

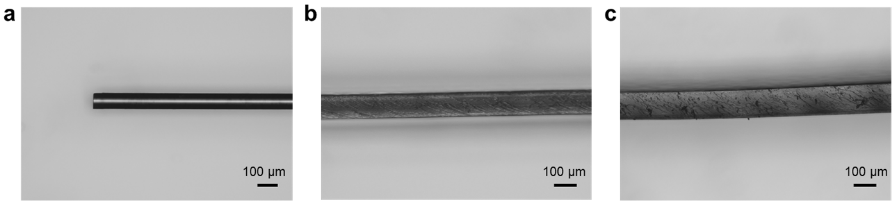

Supplementary Fig. 10 Optical microscope images of an 80 μm optical fibre and actuators based on it. a Optical microscope image of an optical fibre with a diameter of 80 μm. **b** Optical microscope image of an actuator fabricated with an 80 μm optical fibre showing a total thickness of ~100 μm. **c** Optical microscope image of an actuator fabricated with an 80 μm optical fibre showing a total thickness of ~120 μm.

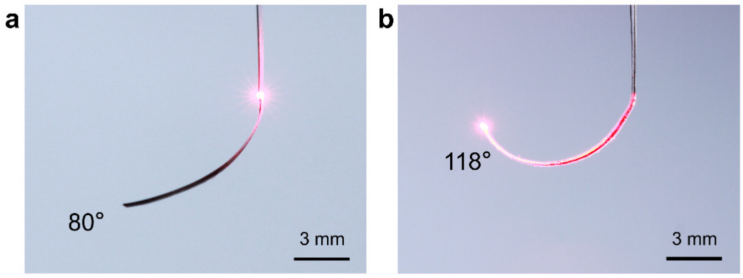

Supplementary Fig. 11 Light-driven actuating performances of actuators fabricated with 80-µm-diameter optical fibre. **a** Photograph showing the light-driven bending of an actuator with a thickness of ~100 µm under 150 mW laser. **b** Photograph showing the light-driven bending of an actuator with a thickness of ~120 µm under 150 mW laser.

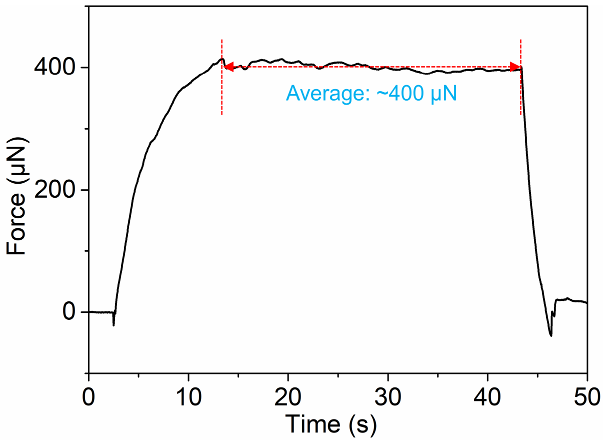

Supplementary Fig. 12 Bending force of an OPA (10 × 0.5 × 0.07 mm^3) over a laser-on and laser-off cycle with a power of 150 mW. The average force is measured to be ~400 μN. Source data are provided as a Source Data file.

Supplementary Table 1 Comparison of performances of photoactuators.

Type of devices	Materials	Bending angle[°]	Energy consumption [mW/cm^2/°]	Response time	Lifting weight ratio	Ref.
Waveguide photoactuators	LCE	~15	15.33 mW/°	~5 s for 15° bending	NA	[1]
	LCE	~17	0.59 mW/°	~1.5 s for 10° bending	NA	[2]
	PNIPAm/NOA 68	~60	4.63 mW/°	~5 s for 50° bending	NA	[3]
	OPA	>270	<0.55 mW/°	1.8 s for 180° bending	71	**This work**
Free-space light-driven photoactuators	PDMS/SIO	~50	10	10 s for 50° bending	NA	[4]
	LCE	90	3.3	0.2 s for 90° bending	Hundreds	[5]
	LCE	115	0.41	23 s for 115° bending	NA	[6]
	LCN	90	0.44	~3 s for 90° bending	NA	[7]
	LCN	90	0.56	0.5 s for 90° bending	NA	[8]
	LCN	115	6.25	30 s for 115° bending	100	[9]
	LCG	~105	3.05	~0.5 s for 75° bending	NA	[10]
	PI/CNT-paraffin	85	1.18	0.87 s for 85° bending	NA	[11]
	CNT-paper/BOPP	~230	0.87	10 s for 230° bending	10	[12]
	PC/SWNT	90	1.11	1 s for 90° bending	NA	[13]
	Chitosan/PDMS-CNT	220	2.27	16s for 220° bending	~4.5	[14]
	VO_2/SWNT	~30	1	~0.02 s for 30° bending	NA	[15]
	PE-BZT-GN	~90	2.33	~2s for 90° bending	20	[16]
	GO/PPy	360	0.35	~5s for 90° bending	~38	[17]
	PMMA/AuNRs-RGO	~80	1	~0.6 s for 60° bending	NA	[18]
	AuNR-PNIPAAM	90	5.6 W	~0.5 s for 90° bending	NA	[19]
	PNIPAM/PPy	70	31.4	5s for 70° bending	~7.8	[20]
	PE/I-MXene	700	0.29	2.1 s for 700° bending	1.7	[21]
	PC/MXene-cellulose	~170	0.47	~6 s for 170° bending	NA	[22]
	WS_2-SA	60	1.83	9 s for 60° bending	500	[23]

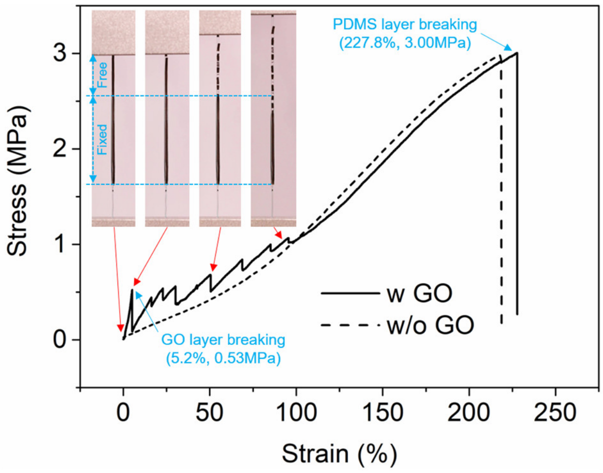

Supplementary Fig. 13 Tensile stress-strain curves of OPAs with or without GO layer. The tensile stress-strain curve of an OPA undergoes a sharp decrease of tensile stress at a strain of ~5.2 %, which is ascribed to the break of GO layer. The inserts show a series of breaking points of GO layer in the strain range of 5 %-100 %, which respectively correspond to the sharp decrease points in the stress-strain curve. With the further increase of the tensile strain, the stress increases until the breaking of PDMS layer, with an elongation of ~227.8 % and a tensile strength of ~3.00 MPa. The tensile stress-strain curve of an OPA without GO shows no stress decrease until the breaking of PDMS-AuNR layer at ~218.6 % strain, which verifies that the decrease points in the tensile stress-strain curve of OPA are due to the breaking of the GO layer. Source data are provided as a Source Data file.

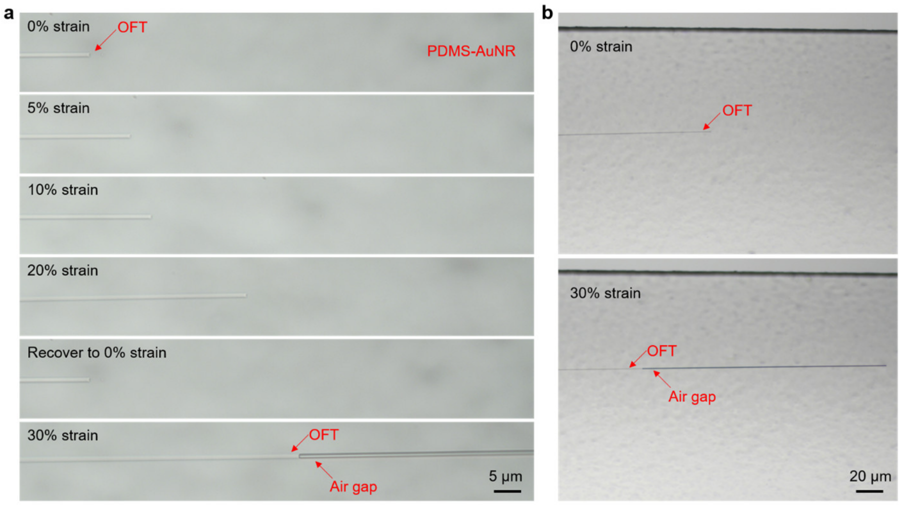

Supplementary Fig. 14 Optical microscope images of OFT embedded in PDMS-AuNR film under different strain. a Optical microscope images of an OFT embedded in a PDMS-AuNR under different strains. There is no delamination between the OFT and PDMS-AuNR matrix under 5 %, 10 % and 20 % strain, and the sample can recover to its initial state after releasing the tensile strain. When the strain is increased up to 30 %, the OFT tip is delaminated from the PDMS-AuNR matrix and an air gap can be observed. **b** Optical microscope images of OFT embedded in PDMS-AuNR matrix before and after 30 % tensile strain.

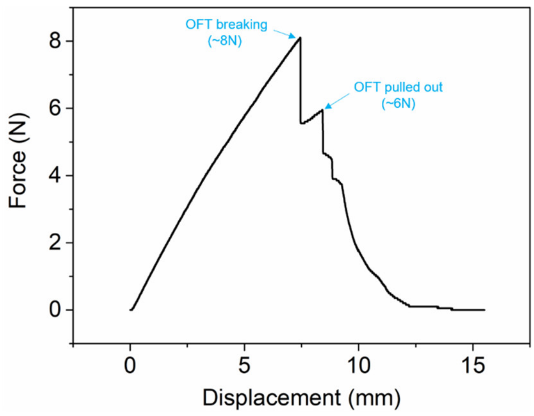

Supplementary Fig. 15 Tensile force for pulling OFT out of PDMS-AuNR matrix. The tensile force for pulling OFT out of PDMS-AuNR matrix decreases sharply after reaching ~8 N, which is due to the breaking of OFT. After that, the tensile force increases again until the OFT starts to be pulled out with a corresponding delaminating force of ~6 N. Source data are provided as a Source Data file.

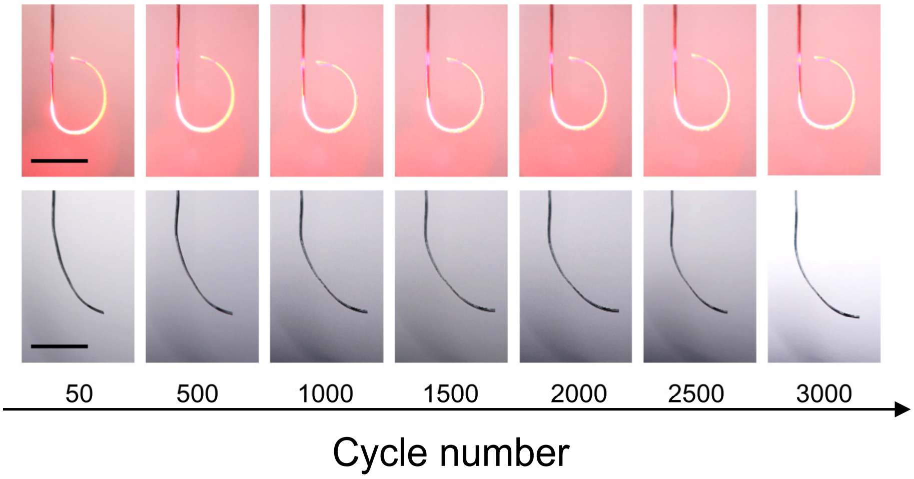

Supplementary Fig. 16 Photographs showing the bending angles of the OPA (10 × 0.5 × 0.07 mm^3) over 3000 cycles of laser-on and laser-off with a power of 100 mW. Scale bar: 5 mm.

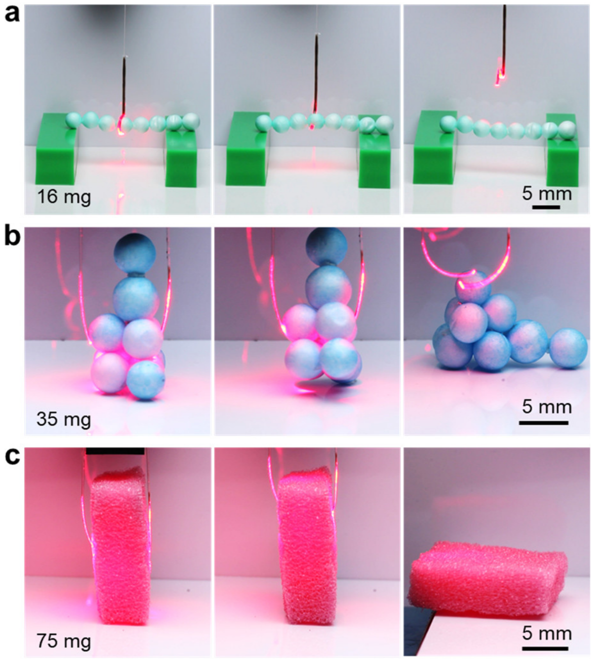

Supplementary Fig. 17 OPA grippers fail to capture too heavy objects. a The one-arm OPA gripper fails to capture 16 mg glued balls. With the switch on of 635 nm laser, the OPA wraps around the 16 mg glued balls. However, when lifted up, the too heavy balls roll over and fall down from the OPA gripper, causing the failure of capturing. **b** The two-arm OPA gripper fails to capture 35 mg glued balls. The too heavy balls cause the unstability during lifting, leading to the failure of capturing. **c** The two-arm OPA gripper fails to capture a 75 mg cuboid. The 75 mg cuboid is too big to be tightly gripped and too heavy to be lifted, causing the failure of capturing.

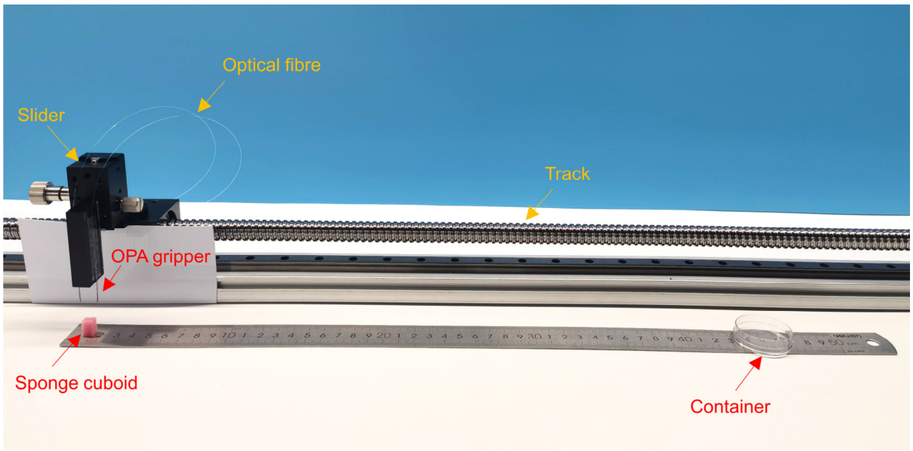

Supplementary Fig. 18 Photograph of the device with a two-arm OPA soft gripper for capturing and moving objects in wide operating area.

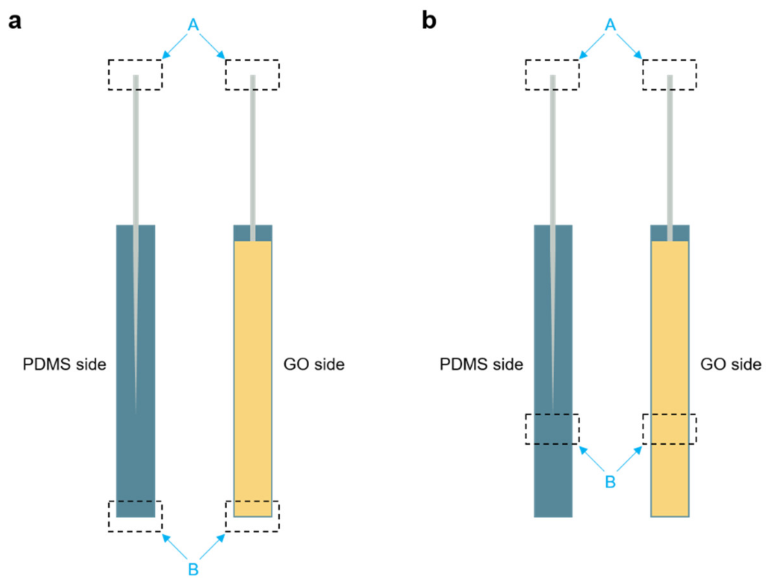

Supplementary Fig. 19 Schematic of different methods for fixing sample in tensile tests. a Method of fixing samples in tensile tests of an OPA and delamination test of an OFT in the PDMS matrix. **b** Method of fixing samples in tensile force for pulling an OFT out of a PDMS matrix. A, B: Fixing positions.

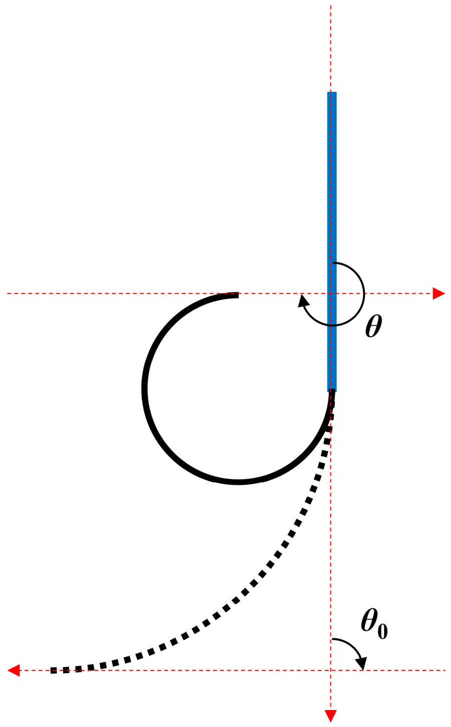

Supplementary Fig. 20 The schematic for calculating bending angle. The θ_0 is the original angle and the θ is the final angle after deformation. The bending angle after deformation is calculated as $\Delta\theta=\theta-\theta_0$.

References

1. Kuenstler, A. S., Kim, H. & Hayward, R. C. Liquid crystal elastomer waveguide actuators. *Adv. Mater.* **31**, 1901216 (2019).
2. Zmyslony, M. *et al.* Optical pliers: Micrometer-scale, light-driven tools grown on optical fibers. *Adv. Mater.* **32**, 202002779 (2020).
3. Zhou, Y., Hauser, A. W., Bende, N. P., Kuzyk, M. G. & Hayward, R. C. Waveguiding microactuators based on a photothermally responsive nanocomposite hydrogel. *Adv. Funct. Mater.* **26**, 5447-5452 (2016).
4. Wang, Y. *et al.* Light-activated shape morphing and light-tracking materials using biopolymer-based programmable photonic nanostructures. *Nat. Commun.* **12**, 1651 (2021).
5. Wani, O. M., Zeng, H. & Priimagi, A. A light-driven artificial flytrap. *Nat. Commun.* **8**, 15546 (2017).
6. Zuo, B., Wang, M., Lin, B. P. & Yang, H. Visible and infrared three-wavelength modulated multi-directional actuators. *Nat. Commun.* **10**, 4539 (2019).
7. Pilz da Cunha, M., Kandail, H. S., den Toonder, J. M. J. & Schenning, A. P. H. J. An artificial aquatic polyp that wirelessly attracts, grasps, and releases objects. *Proc. Natl. Acad. Sci. U.S.A.* **117**, 2004748 (2020).
8. Zeng, H. *et al.* Light-fuelled freestyle self-oscillators. *Nat. Commun.* **10**, 5057 (2019).
9. Lahikainen, M., Zeng, H. & Priimagi, A. Reconfigurable photoactuator through synergistic use of photochemical and photothermal effects. *Nat. Commun.* **9**, 4148 (2018).
10. Shahsavan, H. *et al.* Bioinspired underwater locomotion of light-driven liquid crystal gels. *Proc. Natl. Acad. Sci. U.S.A.* **117**, 5125-5133 (2020).
11. Deng, J. *et al.* Tunable photothermal actuators based on a pre-programmed aligned nanostructure. *J. Am. Chem. Soc.* **138**, 225-230 (2016).
12. Zhou, P., Chen, L., Yao, L., Weng, M. & Zhang, W. Humidity- and light-driven actuators based on carbon

nanotube-coated paper and polymer composite. *Nanoscale* **10**, 8422-8427, (2018).

13. Zhang, X. *et al.* Photoactuators and motors based on carbon nanotubes with selective chirality distributions. *Nat. Commun.* **5**, 2983 (2014).

14. Xu, H. *et al.* An ultra-large deformation bidirectional actuator based on a carbon nanotube/PDMS composite and a chitosan film. *J. Mater. Chem. B* **7**, 7558-7565 (2019).

15. Wang, T., Torres, D., Fernandez, F. E., Wang, C. & Sepulveda, N. Maximizing the performance of photothermal actuators by combining smart materials with supplementary advantages. *Sci. Adv.* **3**, 1602697 (2017).

16. Pan, X., Grossiord, N., Sol, J. A. H. P., Debije, M. G. & Schenning, A. P. H. J. 3D anisotropic polyethylene as light-responsive grippers and surfing divers. *Adv. Funct. Mater.* **31**, 2100465 (2021).

17. Dong, Y. *et al.* Multi-stimuli-responsive programmable biomimetic actuator. *Nat. Commun.* **10**, 4087 (2019).

18. Han, B. *et al.* Plasmonic-assisted graphene oxide artificial muscles. *Adv. Mater.* **31**, 1806386 (2019).

19. Zhao, Y. *et al.* Soft phototactic swimmer based on self-sustained hydrogel oscillator. *Sci. Robot.* **4**, eaax7112 (2019).

20. Luo, R., Wu, J., Dinh, N. D. & Chen, C. H. Gradient Porous Elastic Hydrogels with Shape-Memory Property and Anisotropic Responses for Programmable Locomotion. *Adv. Funct. Mater.* **25**, 7272-7279 (2015).

21. Hu, Y. *et al.* Self-locomotive soft actuator based on asymmetric microstructural ti3c2tx mxene film driven by natural sunlight fluctuation. *ACS Nano* **15**, 5294-5306 (2021).

22. Cai, G., Ciou, J. H., Liu, Y., Jiang, Y. & Lee, P. S. Leaf-inspired multiresponsive MXene-based actuator for programmable smart devices. *Sci. Adv.* **5**, eaaw7956 (2019).

23. Zong, L., Li, M. & Li, C. Bioinspired coupling of inorganic layered nanomaterials with marine polysaccharides for efficient aqueous exfoliation and smart actuating hybrids. *Adv. Mater.* **29**, 1604691 (2017).
