# Supplementary material for: Optical fibre taper-enabled waveguide photoactuators
Source: Nat Commun. 2022 Jan 18;13:363. doi: 10.1038/s41467-022-28021-4 (PMC8766484; doi:10.1038/s41467-022-28021-4)
Supplement: Supplementary file 2 — Description of Additional Supplementary Files [file 41467_2022_28021_MOESM2_ESM.docx]

**Description of Additional Supplementary Files**

**Supplementary Movie 1:** Light-driven bending of OPA.

**Supplementary Movie 2:** Mechanical strength of OPA.

**Supplementary Movie 3:** OPA wind s and unwinds a pipe.

**Supplementary Movie 4:** OPA captures 10 mg small balls.

**Supplementary Movie 5:** OPA captures 12 mg small balls.

**Supplementary Movie 6:** OPA captures 14 mg small balls.

**Supplementary Movie 7:** OPA fails to capture 16 mg small balls.

**Supplementary Movie 8:** OPA captures an ant.

**Supplementary Movie 9:** OPA gripper captures 20 mg small balls.

**Supplementary Movie 10:** OPA gripper captures 25 mg small balls.

**Supplementary Movie 11:** OPA gripper captures 27 mg small balls.

**Supplementary Movie 12:** OPA gripper fails to capture a 35 mg small balls.

**Supplementary Movie 13:** OPA gripper captures a 20 mg cuboid.

**Supplementary Movie 14:** OPA gripper captures a 40 mg cuboid.

**Supplementary Movie 15:** OPA gripper captures a 68 mg cuboid.

**Supplementary Movie 16:** OPA gripper fails to capture a 75 mg cuboid.

**Supplementary Movie 17:** OPA gripper handles a cuboid.
